# Why Far-Red Photons Should Be Included in the Definition of Photosynthetic Photons and the Measurement of Horticultural Fixture Efficacy

**DOI:** 10.3389/fpls.2021.693445

**Published:** 2021-06-22

**Authors:** Shuyang Zhen, Marc van Iersel, Bruce Bugbee

**Affiliations:** ^1^Department of Horticultural Sciences, Texas A&M University, College Station, TX, United States; ^2^Department of Horticulture, University of Georgia, Athens, GA, United States; ^3^Department of Plants, Soils and Climate, Utah State University, Logan, UT, United States

**Keywords:** far-red photons, fixture efficacy, photosynthesis, Emerson Enhancement Effect, extended photosynthetically active radiation

## Introduction

Photons above 700 nm have minimal photosynthetic activity when applied alone (Emerson and Lewis, 1943; McCree, 1971) and have thus been excluded from the definition of photosynthetically active radiation (PAR; 400 to 700 nm). However, those longer-wavelength photons have synergistic activity with photons in the PAR range (Emerson et al., 1957). Recent studies using lasers and LEDs with narrow-band spectra have provided new insights into the photosynthetic value of far-red photons (here defined as 700 to 750 nm). Far-red photons preferentially excite photosystem I (Zhen and van Iersel, 2017), at wavelengths at least up to 732 nm (Zhen et al., 2019). In crop-plant communities, far-red photons elicit photosynthetic activity equal to PAR photons when delivered at up to 30% of the total photon flux (Zhen and Bugbee, 2020a). The quantum yield of plant canopies (per 400 to 750 nm photons) is similar under blue + red or white LEDs with and without a 15% far-red photon substitution (Zhen and Bugbee, 2020b). The definition of photosynthetic photons, and efficacy measurements of horticultural fixtures, need to include far-red photons because this extended range (referred to as ePAR) better predicts photosynthesis.

## Historical Background

Photosynthesis has long been known to be wavelength-dependent (Hoover, 1937; Emerson and Lewis, 1943). At low photon flux densities, McCree (1971) and Inada (1976) found that red photons (600–700 nm) drive photosynthesis more efficiently than green (500–600 nm), followed by blue (400–500 nm) photons. Because green photons penetrate deeper into leaves, more recent studies indicate that at higher photon flux densities red and green photons are used more efficiently than blue photons (Terashima et al., 2009; Liu and van Iersel, 2021). Longer-wavelength far-red photons (above 700 nm), on the other hand, are largely inactive for photosynthesis when applied alone (Emerson and Lewis, 1943; McCree, 1971) and have thus been excluded from the definition of photosynthetically active radiation (PAR; 400–700 nm).

The rapid decline in photosynthetic efficiency at longer wavelengths (above ~685 nm) was first observed by Emerson and Lewis (1943) in green algae (the “red drop”). Over a decade later, the same research group found that the photosynthetic rate under simultaneous illumination with photons above 680 nm and shorter-wavelength light was greater than the sum of the rates from applying each light separately (Emerson et al., 1957). This is now known as the Emerson Enhancement Effect. This enhancement effect among shorter- and longer-wavelength photons was later found to be due to the distinct excitation spectra of the two photosystems—PSI and PSII (Hill and Bendall, 1960; Duysens and Amesz, 1962). While the discovery of the Emerson Enhancement Effect contributed to the identification of PSI and PSII, the significance of the wavelength synergy in photosynthetic efficiency received little attention over the next 50 years and the spectral effects on photosynthesis continued to be studied under monochromatic lights. The main reason for this oversight is the belief that only the photosynthetic efficiency of longer-wavelength photons (~680 nm up to 720 nm) was improved by the supplementation with shorter-wavelength light, rather than a two-way synergistic interaction in which shorter- and longer-wavelength photons improve each other's photosynthetic efficiency; thus, the practical impact of the enhancement effect on photosynthesis was thought to be largely insignificant (Emerson et al., 1957; Myers and Graham, 1963; McCree, 1972a). As a result, the now widely accepted definition of PAR was developed without taking account of synergistic effects on PSI and PSII photochemistry between far-red and shorter-wavelength photons.

This 400–700 nm range was recommended by McCree (1972b) from among the most popular definitions of PAR in use at the time. He concluded that the photon flux density between 400 and 700 nm was “an acceptable definition of photosynthetic flux” for nine commonly used broad-spectrum lights. Interestingly, he found that photosynthetic rates, normalized based on PAR, were highest under a high-pressure sodium light (with most of the light in the red part of the spectrum) and a quartz-iodine light (rich in red and far-red photons). None of the definitions of PAR analyzed by McCree accounted for photons with wavelengths >710 nm. His study did not test whether including far-red photons in the definition of PAR would improve the correlation with the photosynthetic rate.

## Recent Studies Indicate that the Classic Definition of Par Needs to Be Revised

Recent advances in light-emitting diode (LED) technology have enabled researchers to re-visit the Emerson Enhancement Effect and study not only the short-term photosynthetic responses of single leaves under low light but also the long-term responses of plant canopies under higher photon flux densities. Zhen and van Iersel (2017) found that adding supplemental far-red photons from LEDs (peak at 735 nm) to red+blue or white LED light synergistically increased the quantum yield of PSII and leaf photosynthetic rate over a wide range of light intensities (also see Murakami et al., 2018). The enhancement was slightly larger under the red/blue background light than under a warm white LED, probably because the warm white LED light already had 4% far-red photons.

Zhen et al. (2019) studied the effects of photons from 678 to 752 nm using laser diodes that had a narrow spectral output [full width at half maximum (FWHM) of 2–3 nm]. As the wavelength of the photons increased from 678 to 703 nm, they increasingly excited PSI more efficiently than PSII. Photons up to 732 nm significantly enhanced photosynthetic efficiency by exciting PSI, but photons above 752 nm were not effective. There was a gap between 732 and 752 nm because laser diodes were not available in this region.

In a subsequent study, Zhen and Bugbee (2020a) measured the net photosynthetic rate of plant canopies (including leaves, stems, roots; with communities of plants inside 100 L gas exchange chambers) in 14 diverse species and found that photons from far-red LEDs (700–750 nm; peak at 735 nm) were as effective as traditional 400–700 nm photons when applied at up to ~30% of the total photon flux. As expected, far-red photons alone were not effective. Additional far-red photons applied at more than 30% of the total photon flux did not result in further increases in photosynthetic rate. The photosynthetic response to increasing far-red photon flux likely saturates, because under a light composed of mostly shorter-wavelength photons that over-excites PSII, only a certain number of far-red photons are needed to restore the excitation balance between PSI and PSII. An enhancement effect only occurs up to the point at which this balance is reached.

Zhen and Bugbee (2020b) followed this study with a long-term study with lettuce grown under either blue+red or white LEDs, each with and without 15% far-red photons from far-red LEDs. The total photon flux from 400 to 750 nm was equal among spectral treatments. Photon capture and canopy gas exchange were continuously measured, which allowed the analysis of canopy quantum yield (CQY; moles of CO_2_ fixed per mole of absorbed photons). CQY was equal among treatments from planting to harvest, confirming the important role of far-red photons for photosynthesis.

We have studied the effects of far-red photons at the photosystem, leaf, and canopy level, with consistent results at all three scales. Collectively, these findings provide compelling evidence for the photosynthetic value of far-red photons when combined with shorter-wavelength photons, as long as the far-red flux does not exceed about 30% of the total photon flux. This is equal to or higher than the fraction of far-red photons in sunlight under which plants have evolved.

Unfortunately, the Design Lights Consortium (2021), following ASABE Standard S640 (2017), recently decided to not expand the definition of PAR to include far-red photons, despite clear evidence of the photosynthetic efficacy of those photons. Design Lights Consortium kindly cites our research in their decision, but we do not believe that our research was interpreted accurately. One concern raised by Design Lights Consortium (2021) is that the enhancement effect may depend on the spectrum of the background light. As we explained earlier, the larger enhancement effect of far-red photons with red+blue background light compared to that under a white background light was most likely because the white light already had ~4% far-red photons. In addition to this, Zhen and Bugbee (2020b) found that the value of far-red photons was equal with either blue+red or white LEDs (contained ~1% far-red photons). As far as we know there is no experimental evidence suggesting that the spectral composition in the 400–700 nm range affects the magnitude of the enhancement effect.

Design Lights Consortium also argued that the data presented in Zhen and van Iersel (2017) suggested that the enhancement response diminished at higher background light intensities, thus the response is non-linear and the prediction of photosynthesis with the extended PAR range would be difficult. This was a misinterpretation, since Zhen and van Iersel (2017) added the same intensity of far-red photons to varying background light intensities between 400 and 700 nm and found that the absolute increase in net photosynthetic rate was similar at all background light intensity levels; it was the *percent* increase in net photosynthetic rate that diminished at higher background light intensities, which is expected.

In addition, the Design Lights Consortium (2021) also mistakenly described the equal canopy photosynthetic rate elicited by far-red substitution in Zhen and Bugbee (2020a) as an “additive” response without any enhancement effect. In fact, the data clearly show that the far-red photons alone had little photosynthetic activity. However, when added to a background of 400–700 nm photons, far-red photons increased photosynthesis equal to the addition of traditional PAR photons.

Lastly, the Design Lights Consortium (2021) raised concern about the spectral response of far-red photons. Zhen et al. (2019) found that photons with wavelengths from ~700 to 732 nm were similarly effective in enhancing photochemical efficiency, but photons above 752 nm were not effective. Zhen and Bugbee (2020a) added photons from three far-red LEDs with peak output at 711, 723, and 746 nm, respectively, to a background of red+blue light and found that the increase in photosynthesis in response to the added far-red decreased with increasing peak wavelength of the far-red LEDs. About 29% of the total photons emitted by the 711 nm-centered LED was below 700 nm, and about 24% of the total photons emitted by the 746 nm-centered LED was above 750 nm. Since these LEDs had a relatively broad emission spectrum (FWHM ranging from 18 to 24 nm), it is difficult to draw a definitive conclusion on the importance of the spectral effect of far-red photons. The argument that precise knowledge of the spectral response of photosynthesis to far-red photons is required before far-red can be included in the definition of PAR is inconsistent with the current definition of PAR: it is well-known that photons of different wavelengths within the 400–700 nm range elicit different photosynthetic responses. That has not stopped the widespread adoption of photons within the 400–700 nm range as the definition of PAR. Defining PAR as the photon flux density between 400 and 750 nm may not be perfect, but will more accurately reflect the photosynthetic activity of photons and better correlate with plant growth than the current definition. To keep the traditional PAR definition and the new definition separate, we have begun using the term extended PAR (ePAR) to refer to the sum of photons between 400 and 750 nm.

Since the efficacy of horticultural fixtures (μmoles of photons per joule) is calculated as photosynthetic photons/energy use, the efficacy of fixtures that include far-red photons will be counted as lower than fixtures that do not emit far-red photons. This occurs with lower color temperature white LEDs and especially fixtures with far-red LEDs. Fixture efficacy is used by energy efficiency programs to determine fixture eligibility for rebate or incentive programs. Until the definition of PAR is expanded to include far-red photons (ePAR, 700–750 nm), lighting manufacturers will be discouraged from including far-red LEDs in fixtures. Changing the definition from PAR to ePAR and including photons from 400 to 750 nm will facilitate the development of fixtures with higher efficacy and encourage innovation (Kusuma et al., 2020). As more supporting data from more laboratories becomes available, we expect that the ePAR definition will replace the current definition of PAR. The ePAR definition should indicate that the far-red fraction cannot exceed about 30% of the total photon flux from 400 to 750 nm or 40% of the photon flux from 400 to 700 nm.

## Practical Limitations

Far-red photons typically cause significant stem, leaf, and/or petiole elongation, which will likely limit the maximum fraction of far-red photons to less than about 20% of the total photon flux for most crops. Because of these powerful effects, we recommend that LED manufacturers clearly indicate the fraction of far-red photons in fixture specifications [(700–750 nm)/(400–750 nm)].

## Author Contributions

This opinion piece originated from discussions among all authors. SZ wrote the first draft with input from MvI and BB. All authors revised the manuscript and have approved the manuscript.

## Conflict of Interest

The authors declare that the research was conducted in the absence of any commercial or financial relationships that could be construed as a potential conflict of interest.
